# Early Intra-Aortic Balloon Pump Support and In-Hospital Mortality in Patients with LV Dysfunction and Cardiogenic Shock Complicating AMI

**DOI:** 10.3390/jcm15031046

**Published:** 2026-01-28

**Authors:** Kina Jeon, Bum Sung Kim, Woo Jin Jang, Ki Hong Choi, Jeong Hoon Yang, Sung Hea Kim, Cheol Woong Yu, Jin-Ok Jeong, Hyun-Jong Lee, Hyeon-Cheol Gwon, Haseong Chang, Hyun-Joong Kim

**Affiliations:** 1 Division of Cardiology, Department of Medicine, Konkuk University Medical Center, Seoul 05030, Republic of Korea20150056@kuh.ac.kr (B.S.K.); shkim@kuh.ac.kr (S.H.K.); 2 Division of Cardiology, Department of Internal Medicine, Chung-Ang University Hospital, Chung-Ang University College of Medicine, Seoul 06973, Republic of Korea; wj78914@gmail.com; 3 Division of Cardiology, Department of Medicine, Heart Vascular Stroke Institute, Samsung Medical Center, Sungkyunkwan University School of Medicine, Seoul 06351, Republic of Korea; cardiokh@gmail.com (K.H.C.); jhysmc@gmail.com (J.H.Y.); hcgwon@naver.com (H.-C.G.); 4 Division of Cardiology, Department of Internal Medicine, Korea University Anam Hospital, Seoul 02841, Republic of Korea; ycw717@naver.com; 5 Division of Cardiology, Department of Internal Medicine, Chungnam National University Hospital, Daejeon 35015, Republic of Korea; jojeong@cnu.ac.kr; 6 Division of Cardiology, Department of Medicine, Sejong General Hospital, Bucheon 14754, Republic of Korea; untouchables00@hanmail.net; 7 Department of Cardiology, Hyundae Hospital, 21 Bonghyeon-ro, Jinjeop-eup, Namyangju 12013, Republic of Korea

**Keywords:** cardiogenic shock, intra-aortic balloon pump, timing of therapy

## Abstract

**Background**: Despite advancements in mechanical support (MCS) devices, the mortality rate for patients with cardiogenic shock remains high. This study aimed to evaluate the efficacy of early intra-aortic balloon pump (IABP) support compared to medical therapy in patients with cardiogenic shock (CS) due to acute myocardial infarction (AMI) (AMI-CS) resulting in severe left ventricular (LV) systolic dysfunction. **Methods**: We analyzed the RESCUE I registry (NCT02985008), a multicenter cohort of 1247 cardiogenic shock patients. A total of 192 patients with AMI-CS with LVEF ≤ 35% received either medical therapy (*n* = 105) or IABP support (*n* = 87) after shock development. The primary outcome was in-hospital mortality. Then, we compared mortality in early IABP initiation (shock-to-IABP < 2 h) to medical therapy. **Results**: The overall in-hospital mortality rate was 42.2%. While the difference in mortality rates between the medical therapy group and the IABP group was not statistically significant (47.6% vs. 35.6%, respectively, *p* = 0.094), a reduction in mortality was observed when IABP support was initiated within 2 h of shock onset (32.0% vs. 47.6%, *p* = 0.036). Furthermore, the need for advanced MCS was reduced in the IABP group compared to the medical group (4.6% vs. 22.9%, respectively, *p* < 0.001). **Conclusions**: In patients with AMI-CS and severe LV dysfunction, early IABP support initiated within 2 h of shock onset was associated with lower in-hospital mortality and reduced need for advanced MCS. These findings highlight the critical importance of timing rather than routine use, supporting a selective strategy for early IABP.

## 1. Introduction

Cardiogenic shock (CS) from acute myocardial infarction (AMI) (AMI-CS) continues to carry mortality rates exceeding 40–50% despite advances in revascularization and hemodynamic support [1,2]. Since the landmark SHOCK trial demonstrated survival benefit with early revascularization [2], subsequent investigations have focused on mechanical circulatory support (MCS) devices to augment cardiac output and end-organ perfusion. Among these, the intra-aortic balloon pump (IABP) has been the most widely used modality for over five decades due to its relative technical simplicity and favorable safety profile. IABP is a percutaneously inserted, temporary MCS device consisting of a balloon-tipped catheter positioned in the descending thoracic aorta that inflates at diastole and deflates just before systole, thereby augmenting diastolic aortic pressure and coronary perfusion, reducing left ventricular (LV) afterload and myocardial oxygen consumption, and providing modest improvement in cardiac output [3].

The IABP-SHOCK II trial [4] randomized 600 patients with AMI-CS to IABP versus conventional therapy, finding no 30-day and 6-year mortality benefit [5]. Consequently, contemporary guidelines assign a Class III recommendation against routine IABP use in AMI-CS. However, subsequent analyses have revealed important nuances.

Emerging evidence suggests that timing of MCS implementation is a critical determinant of outcomes. Recent registry data demonstrate that early initiation of MCS is associated with improved survival compared to delayed implementation [6], whether it be IABP or more advanced devices like extracorporeal membrane oxygenation (ECMO) and Impella [7,8]. The concept of “right-sizing” MCS to patient phenotype—matching device capability to underlying pathophysiology—has gained traction as an alternative to uniform application strategies. In a recent cohort, MCS reduced 30-day mortality only when LV ejection fraction (LVEF) was ≤20%, while no benefit or potential harm was seen at higher LVEF, supporting LV function-based selection for support [9,10]. Another analysis demonstrated that IABP implantation in early cardiogenic shock remarkably reduced 30-day mortality and prevented shock progression [11], supporting selective, timing-dependent use by using the SCAI shock classification system—a framework for risk stratification and appropriate MCS selection based on shock severity [12].

Based on these insights, we hypothesized that selective application of IABP to patients with AMI-CS characterized by severe LV systolic dysfunction, when initiated early in the shock course, would demonstrate survival benefit compared to medical therapy alone. Using the multicenter RESCUE I registry, we sought to evaluate the efficacy of early IABP support in this specifically defined high-risk population [9,10,13].

## 2. Materials and Methods

### 2.1. Study Protocol

RESCUE I (REtrospective and prospective observational Study to investigate Clinical oUtcomes and Efficacy of left ventricular assist device for Korean patients with CS, NCT02985008) is a multi-center registry for CS. This study was conducted at 12 tertiary care centers in Korea and recruited 1247 patients over the age of 19. To be included for the registry, patients had to have a systolic blood pressure < 90 mmHg for 30 min or require inotropic or vasopressor support to achieve a systolic blood pressure > 90 mmHg, or signs of impaired organ perfusion and pulmonary congestion. Patients who had out-of-hospital cardiac arrest, other types of shock, or refused treatment were excluded. Tertiary hospitals participating in the RESCUE I registry aimed to provide guideline-directed medical therapy for AMI and CS; early revascularization, dual antiplatelet therapy, anticoagulation, and hemodynamic support were all according to ACC/AHA and ESC recommendations during the study period. IABP insertion was at the discretion of the treating interventional cardiologist. In patients presenting with CS or progressing toward shock, IABP was generally placed prior to PCI when feasible to stabilize hemodynamics before revascularization.

The RESCUE I registry collected patient demographic, in-hospital management, laboratory, procedural, and outcome data through web-based case report forms managed by independent clinical research coordinators. If needed, additional information was obtained from medical records or via telephone communication. Institutional review board approval was obtained at all participating centers. The requirement for informed consent was waived by the Institutional Review Boards for patients enrolled retrospectively, and informed consent was obtained for all prospectively enrolled patients.

To identify a homogeneous patient subset who would benefit from IABP support, only patients with AMI complicated by CS due to LV dysfunction were included for analysis. These patients were then grouped based on their initial management after CS development, either medical therapy alone or IABP support. The primary outcome was in-hospital mortality. In depth, we specifically evaluated how medical therapy group compared to IABP according to timing by comparing in-hospital mortality across predefined shock-to-IABP intervals (<15 min, <1 h, <2 h, <3 h, and 3–6 h). Patients who received ECMO within 6 h of developing CS were excluded from the analysis. Because of the modest sample size and the limited number of patients within each timing stratum, we did not apply formal multiplicity adjustments or perform data-driven cut-point optimization; mortality across predefined shock-to-IABP intervals was compared descriptively, and the 2 h cutoff was treated as exploratory rather than as a formally selected optimal threshold.

Comprehensive transthoracic echocardiography was performed with commercially available equipment (Vivid 7, GE Medical Systems, Milwaukee, WI, USA; Acuson 512, Siemens Medical Solution, Mountain View, CA, USA; or Sonos 5500, Philips Medical System, Andover, MA, USA). LVEF was assessed by biplane Simpson’s rule using manual tracing of digital images.

**Figure 1:**
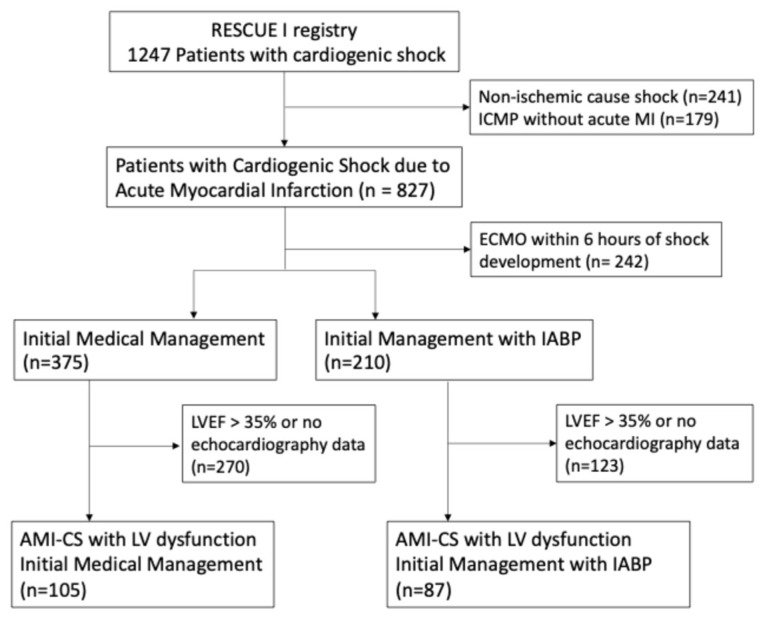
Flow chart for study. ICMP, ischemic cardiomyopathy; MI, myocardial infarction; ECMO, extracorporeal membrane oxygenation; IABP, intra-aortic balloon pump; LVEF, left ventricular ejection fraction; AMI, acute myocardial infarction; AMI-CS, cardiogenic shock from acute myocardial infarction; LV, left ventricle.

Out of 1247 initially enrolled cardiogenic shock patients, 241 patients with non-ischemic CS and 179 patients with known ischemic cardiomyopathy but without AMI presentation were excluded, leaving 827 AMI-CS patients. Among these, 242 patients underwent early implementation of ECMO within 6 h of shock development. Patients with documented LV dysfunction, defined as an echocardiography-assessed LVEF of 35% or less, were further analyzed, resulting in a final 192 patients. Of these, 105 patients were initially managed medically, while 87 patients received IABP support within 6 h of shock development (Figure 1).

### 2.2. Statistical Analysis

Categorical variables are presented as numbers and relative frequencies, and their group differences were compared using the chi-square test. Continuous variables were compared using the Student *t*-test or Mann–Whitney U test, as appropriate, and reported as either the mean ± SD or the median and interquartile range. All probability values were two-tailed, and *p* values < 0.05 were considered statistically significant. For the descriptive and univariable comparisons, each analysis was performed using all available data for the variables included, and the denominators reflect the number of patients with non-missing values. Multivariable logistic regression analysis was performed to identify independent factors associated with the outcome, and variables with clinical relevance in univariable analysis were entered into the model. Statistical analyses were performed using R Statistical Software (version 4.1.3; R Foundation for Statistical Computing, Vienna, Austria).

### 2.3. AI Statement

During the preparation of this manuscript, the authors used Perplexity for the purposes of reference search and descriptive rephrasing. The authors have reviewed and edited the output and take full responsibility for the content of this publication.

## 3. Results

### 3.1. Baseline Characteristics

Baseline characteristics are shown in Table 1. In comparison to the medical group, the IABP group had a higher proportion of male patients (63.8% vs. 79.3%, *p* = 0.019) and a higher mean weight (61.8 ± 12.1 vs. 65.9 ± 13.2, *p* = 0.028) and body mass index (23.3 ± 3.5 vs. 23.8 ± 3.6, *p* = 0.02), respectively. The majority of patients presented with ST segment elevation myocardial infarction (STEMI) (60.0% in the medical group, 58.6% in the IABP group, *p* = 0.846). Most patients had left main (LM) or left anterior descending coronary artery (LAD) disease (93.0% in the medical group, 96.5% in the IABP group, *p* = 0.290), which may have contributed to LV pump failure as an etiology of CS. The mean LVEF was similar between the medical group and IABP group (26.4 ± 7.2% vs. 27.0 ± 6.0%, respectively, *p* = 0.530). Lactate levels were elevated above 5.0 in the medical group and IABP group (6.3 ± 4.7 vs. 5.8 ± 4.1, *p* = 0.472). The median shock-to-IABP interval was 19 min (IQR 0–74 min), with a mean of 51 min. Shortest time from shock to IABP was 0 min, meaning IABP was inserted at the time of shock.

### 3.2. Early IABP and Mortality

The in-hospital mortality rate among patients with AMI-CS and LV systolic dysfunction was high, with an overall rate of 42.2% (Table 2). There was no statistically significant difference inin-hospital mortality rates between the medical group (47.6%. 95% CI of 37.8–57.6%) and the IABP group (35.6%, 95% CIs of 25.6–46.6%) (*p* = 0.094). For this primary comparison, we additionally calculated the unadjusted risk ratio, which was 0.75 (95% CI 0.53–1.06), indicating no statistically significant difference in mortality between groups.

For in-hospital mortality, variables with clinical relevance were entered into a multivariable logistic regression model (Table 3). Among 192 eligible patients, 72 with missing key covariates (including 67 without lactic acid measurements and others without troponin, etc.) were excluded, resulting in 120 patients in the final multivariable analysis. In the multivariable logistic regression included age, sex, DM, previous MI, dyslipidemia, lactic acid, and IABP strategy, etc. DM and previous MI remained independent predictors of in-hospital mortality, whereas dyslipidemia was independently associated with lower in-hospital mortality. IABP use was not independently associated with in-hospital mortality.

**Figure 2:**
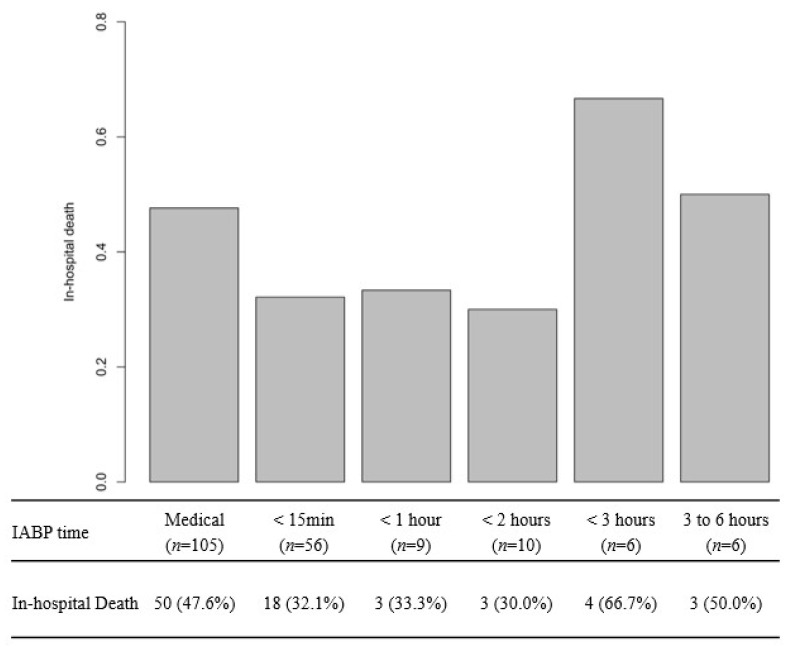
In-hospital mortality according to initiation of IABP.

As shown in Figure 2, it is demonstrated that the mortality rate increased for patients who received IABP support beyond 2 h of shock onset. The mortality rate for patients who received IABP support within the first 15 min, 1 h, and 2 h was 32.1%, 33.3%, and 30.0%, respectively. However, when IABP support began after 2 h of shock onset, the mortality rate surpassed 50%.

A closer look at in-hospital mortality is presented in Table 4. In-hospital mortality was 47.6% (95% CI 37.8–57.6) in the medical group and 32.0% (95% CI 21.7–43.8) among patients receiving IABP within 2 h of shock onset (*p* = 0.036). Compared with patients receiving medical therapy, those managed with IABP within 2 h had an unadjusted risk ratio for in-hospital mortality of 0.77 (95% CI 0.61–0.98, *p* = 0.04). The length of intensive care unit (ICU) stay, rate of continuous renal replacement therapy (CRRT), and incidence of stroke were similar between the two groups. The vasoactive inotropic score was significantly lower in the IABP group compared to the medical group (35.0 vs. 22.7, *p* = 0.002).

Furthermore, 22.9% of patients in the medical group received ECMO support, which was implemented as a salvage measure after initial failed medical stabilization. Unfortunately, the mortality rate in this ECMO group for salvage measure was high, reaching up to 83.3%. Moreover, in cases where IABP support failed to save patients, ECMO was inserted in 4 patients, all of whom failed to survive.

### 3.3. Factors That Favor IABP Support over Medical Treatment

**Figure 3:**
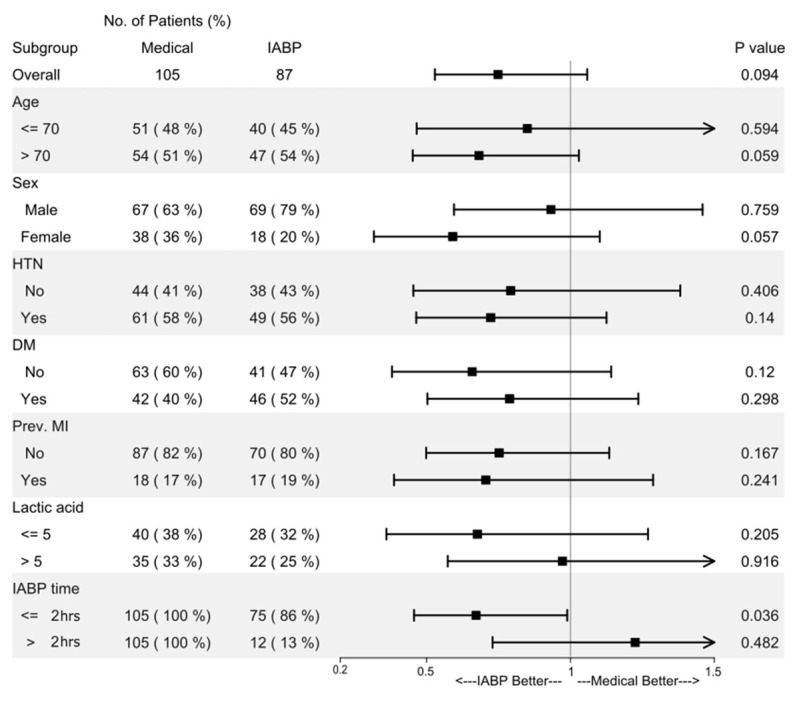
Subgroup analysis of factors favoring IABP vs. medical therapy. IABP, intra-aortic balloon pump; HTN, hypertension; DM, diabetes mellitus; prev., previous; MI, myocardial infarction.

A subgroup analysis was conducted to investigate factors favoring IABP support over medical treatment (Figure 3). The patients were divided into groups according to age (>70 years), gender, presence of hypertension or diabetes mellitus, previous MI, lactic acid level (>5), and the duration from shock to IABP support. The only factor that demonstrated a survival benefit with IABP management was the time interval between shock onset and IABP placement being within 2 h.

## 4. Discussion

This contemporary analysis from the RESCUE I registry demonstrates that in patients with AMI-CS and severe LV systolic dysfunction, compared to medical therapy alone, early IABP support initiated within 2 h of shock was associated with lower in-hospital mortality and less use of advanced MCS compared with medical therapy alone. Conversely, delayed IABP implementation beyond 2 h showed no survival benefit, highlighting the critical importance of timely intervention. These findings emphasize the crucial role of early and appropriate MCS selection in the management of cardiogenic shock.

This observation provides crucial context for the results of IABP-SHOCK II trial [3]. The trial had placed IABP after revascularization in 87% of patients, which may have introduced systematic delay, with median time from randomization to IABP insertion of approximately 2 h. Our data suggests this delay could have obscured benefit in patients who might have responded to earlier support. Furthermore, IABP-SHOCK II trial [4] included more than 40% patients with cardiac arrest, a population in whom IABP’s mechanism requiring intrinsic cardiac pulsation may be less effective. By focusing specifically on patients with severe LV systolic dysfunction, who are most likely to benefit from LV afterload reduction and augmentation of coronary perfusion, we targeted a subgroup that physiology and contemporary reviews identify as the most appropriate candidates for IABP-based LV unloading [10,14,15].

Although the multivariable logistic regression did not demonstrate a statistically significant association between IABP use and in-hospital mortality, this analysis should be interpreted with caution because it included only 120 of the 192 eligible patients after exclusion of cases with missing covariates. This substantial reduction in sample size not only decreases statistical power but may also introduce selection bias, as patients with complete data might differ systematically from those excluded, limiting the ability of the model to fully represent the overall AMI-CS cohort. In this context, the absence of an independent association between IABP and mortality should not be over-interpreted as evidence of no effect but rather viewed as a complementary, hypothesis-generating finding alongside the primary unadjusted and stratified analyses.

In contrast, dyslipidemia emerged as an independent predictor of lower in-hospital mortality in the multivariable model, which may reflect a protective effect of chronic statin therapy rather than dyslipidemia alone. Patients with documented dyslipidemia are more likely to receive long-term statins and other guideline-directed preventive therapies, potentially conferring better baseline vascular health and myocardial resilience. However, because information on statin dose, adherence, and treatment duration was not available, this apparent ‘protective’ association should be interpreted as a marker of prior preventive care and residual confounding, rather than proof of a direct causal effect.

### 4.1. The Importance of Timing in Mechanical Circulatory Support

The importance of timing in MCS implementation is recognized across device types [16]. Studies of percutaneous ventricular assist devices have shown that earlier initiation correlates with better outcomes [17]. A meta-analysis demonstrated that Impella significantly reduces 6-month all-cause mortality in patients with AMI-CS compared to standard care, though this benefit is offset by increased major bleeding, limb ischemia, and sepsis risks [18]. These findings extend the principle to IABP, suggesting that the window of opportunity for effective MCS is narrow and closes as shock progresses to multi-organ failure.

The observation that delayed IABP beyond 2 h eliminated survival benefit, suggests that patients deteriorating despite initial therapy may require escalation to higher-level MCS such as ECMO or Impella rather than continued IABP delay. A recent analysis demonstrated that IABP insertion before primary percutaneous coronary intervention (PPCI), despite longer door-to-balloon times, was associated with improved in-hospital and 6-month survival compared to post-PPCI IABP insertion, further supporting the paradigm of early intervention [19].

### 4.2. Mechanism of IABP Benefit in Early Cardiogenic Shock

The mechanism of IABP benefit in early AMI-CS likely involves multiple pathways. By reducing LV afterload, IABP decreases myocardial oxygen demand in the setting of acute ischemia and reperfusion injury [5]. Augmentation of diastolic pressure improves coronary perfusion pressure, potentially enhancing microvascular flow to the infarct zone and border region. The observed reduction in vasoactive-inotropic scores in our early IABP group suggests successful hemodynamic stabilization with less pharmacologic support, which may independently improve outcomes by reducing catecholamine-mediated myocardial injury and arrhythmogenesis [20].

Our finding that early IABP reduced salvage ECMO requirements from 22.9% to 4.6%, compared to medical therapy alone, has important clinical and economic implications. Salvage ECMO after failed medical therapy carries prohibitively high mortality (more than 80% in our cohort). While ECMO provides superior hemodynamic support, it is consistently associated with higher rates of major complications such as bleeding, limb ischemia, and stroke compared with less invasive devices. Because IABP generally carries a lower complication burden than Impella or ECMO, effective shock management with early IABP in appropriately selected patients may translate into better overall outcomes by stabilizing hemodynamics without immediately exposing patients to the risks of more aggressive support. In this context, early IABP in patients with early stages of shock (roughly corresponding to SCAI B–C) might help prevent progression to refractory shock that would otherwise necessitate escalation to ECMO, whereas patients presenting in more advanced stages (SCAI D–E) are likely to require early high-level support such as ECMO regardless.

In interpreting the lower rate of ECMO implantation in the early IABP group, it is important to recognize that ECMO represents a center- and operator-dependent escalation decision and a potential mediator in the IABP treatment pathway; thus, differences in ECMO use may largely reflect institutional ‘strategic differences’ in shock management rather than a direct measure of disease improvement or intrinsic IABP efficacy.

### 4.3. Clinical Implications

Our findings support a paradigm shift from “routine vs. no IABP” to “right device, right patient, right time.” For patients with AMI-CS and severe LV dysfunction, early IABP within 2 h of shock onset may be considered rather than the recommendation against routine use. Implementation requires standardized shock protocols with rapid identification of eligible patients, immediate availability of IABP equipment and trained personnel, and integrated decision-making.

### 4.4. Limitations

This study has inherent limitations for being a registry analysis, mainly composed of retrospective data. Unmeasured confounders may influence results despite attempts at adjustment. Patients selected for IABP may have differed in subtle but important ways from those managed medically. The study lacks data on the timing of the IABP placement in accordance with PCI, which could influence outcomes. Medical therapy, in terms of dose and duration were not described in detail, lacking evidence in this manner. Also, our cohort includes relatively few patients with IABP placed between >2 h group, potentially limiting power to detect nuances in the timing-mortality relationship. Although immortal time bias is an inherent concern in time-dependent observational comparisons, the short 2 h exposure window relative to the overall in-hospital course suggests that its impact on our findings is likely limited, but it cannot be completely ruled out. Furthermore, because of the modest sample size, we did not perform formal multiplicity adjustments or more complex, data-driven cut-point optimization, which would have been statistically underpowered and prone to overfitting in this cohort; consequently, our choice of the 2 h cutoff should be regarded as exploratory and requires confirmation in larger, prospective studies.

With the development of shock severity stratification (SCAI), it would have been efficient to evaluate with the widespread SCAI classification. However, the RESCUE I registry was initiated before SCAI was proposed, so reliable retrospective SCAI staging is not possible with the available dataset. Also, the single-country Korean population may limit generalizability to other healthcare systems. Lastly, we did not have data on long-term outcomes beyond hospital discharge.

## 5. Conclusions

In patients with AMI-CS and severe LV systolic dysfunction, early IABP support within 2 h was associated with lower in-hospital mortality and reduced need for advanced MCS compared with medical therapy alone. The benefit is lost with delayed implementation, emphasizing that timing is critical. These results support selective early IABP use in AMI-CS with severe LV dysfunction.

## Figures and Tables

**Table 1:** Baseline Patient Characteristics.

	Medical (*n* = 105)	IABP (*n* = 87)	*p*-Value
Age (years)	71.0 (61.0–77.0)	71.0 (62.0–79.5)	0.505
Male	67 (63.8%)	69 (79.3%)	0.019
Height (cm)	163.8 ± 9.2	165.6 ± 8.1	0.161
Weight (kg)	61.8 ± 12.1	65.9 ± 13.2	0.028
BMI (Body Mass Index, kg/m^2^)	23.3 ± 3.5	23.8 ± 3.6	0.02
BSA (Body Surface Area, m^2^)	1.7 ± 0.2	1.7 ± 0.2	0.63
Comorbidities			
Hypertension	61 (58.1%)	49 (56.3%)	0.805
Diabetes mellitus	42 (40.0%)	46 (52.9%)	0.075
Dyslipidemia	32 (30.5%)	35 (40.2%)	0.158
Chronic kidney disease	12 (11.4%)	14 (16.1%)	0.347
Peripheral arterial occlusive disease	3 (2.9%)	6 (6.9%)	0.187
Prior MI	18 (17.1%)	17 (19.5%)	0.668
Prior cerebrovascular event	12 (11.4%)	11 (12.6%)	0.796
Current smoker	33 (31.4%)	28 (32.2%)	0.911
Laboratory			
Hemoglobin (g/dL)	12.2 ± 2.5	12.9 ± 2.2	0.057
Platelet (×10^3^/µL)	211.7 ± 75.3	222.1 ± 68.1	0.320
Total bilirubin (mg/dL)	0.9 ± 0.8	0.9 ± 0.9	0.901
Aspartate transaminase (U/L)	407.9 ± 1511.9	179.5 ± 506.7	0.149
Alanine transaminase (U/L)	196.8 ± 504.1	90.1 ± 309.6	0.075
Serum creatinine (mg/dL)	1.7 ± 1.5	1.7 ± 1.9	0.974
Glucose (mg/dL)	237.4 ± 133.9	241.8 ± 134.2	0.822
Lactate (mmol/L)	6.3 ± 4.7	5.8 ± 4.1	0.472
Troponin I (ng/mL)	92.4 ± 204.6	39.8 ± 96.1	0.023
CK-MB (ng/mL)	248.5 ± 531.3	355.5 ± 1002.9	0.372
LM or LAD disease	93 (93.0%)	83 (96.5%)	0.290
STEMI	63 (60.0%)	51 (58.6%)	0.846
Left ventricular ejection fraction (%)	26.4 ± 7.2	27.0 ± 6.0	0.530
Shock-to-IABP time (median, minutes)		19 (IQR 0–74)	

Values are mean ± standard deviation or *n* (%). Normal ranges are as follows; hemoglobin: male 13.5–17.5 g/dL, female 12.0–15.5 g/dL; platelet 150–400 × 10^3^/µL; total bilirubin 0.3–1.2 mg/dL; AST 10–40 U/L; ALT 7–40 U/L; creatinine 0.6–1.2 mg/dL; fasting glucose 70–115 mg/dL; lactate 0.5–2.2 mmol/L; troponin I < 0.04 ng/mL; CK-MB < 5–10 ng/mL. IABP, intra-aortic balloon pump; MI, myocardial infarction; CK-MB, creatine kinase-myocardial band; LM, left main coronary artery; LAD, left anterior descending coronary artery; STEMI, ST segment elevation myocardial infarction.; IQR, interquartile range.

**Table 2:** In-hospital death according to medical treatment or IABP support.

	Total (*n* = 192)	Medical (*n* = 105)	IABP (*n* = 87)	*p*-Value
In-hospital Death	81 (42.2%)	50 (47.6%)	31 (35.6%)	0.094
ECMO implantation	28 (14.6%)	24 (22.9%)	4 (4.6%)	<0.001
In-hospital Death	24 (85.7%)	20 (83.3%)	4 (100%)	0.378
Length of ICU stay	7.0 (3.0–12.5)	6.0 (2.0–14.0)	7.0 (4.0–12.0)	0.476
CRRT	41 (21.4%)	27 (25.7%)	14 (16.1%)	0.105
Stroke	3 (1.6%)	1 (1.0%)	2 (2.3%)	0.454
Vasoactive Inotropic Score	30.0 (10.2–80.0)	35.0 (16.5–90.0)	22.7 (10.0–54.7)	0.002

Values are *n* (%) or median (interquartile range). IABP, intra-aortic balloon pump; ECMO, extracorporeal membrane oxygenation; ICU, intensive care unit; CRRT, continuous renal replacement therapy.

**Table 3:** Multivariable logistic regression analysis for in-hospital mortality.

	Adjusted OR	95% CI	*p*-Value
Age (per year)	1.01	0.96–1.07	0.644
Male sex	0.67	0.15–3.03	0.592
Height (cm)	0.96	0.88–1.05	0.402
ICU admission weight (kg)	0.99	0.94–1.05	0.846
Hypertension	0.42	0.15–1.12	0.095
Diabetes mellitus	2.94	1.07–8.07	0.037
Dyslipidemia	0.32	0.11–0.89	0.032
Chronic kidney disease	2.75	0.69–11.0	0.150
Peripheral arterial occlusive disease	0.63	0.10–4.11	0.628
Previous myocardial infarction	4.07	1.19–13.9	0.025
Previous cerebrovascular event	0.58	0.11–3.02	0.514
Current smoking	0.36	0.11–1.10	0.084
Lactic acid	1.09	0.98–1.22	0.122
Peak troponin-I	1	0.997–1.003	0.803
Peak CK-MB	1	0.999–1.006	0.074
Vasoactive-Inotropic Score	1	0.999–1.003	0.153
MI type (STEMI vs. NSTEMI)	1.48	0.57–3.82	0.423
IABP use	0.77	0.28–2.12	0.607

Values adjusted odds ratios (OR) with 95% confidence intervals (CI) derived from a multivariable logistic regression model. ICU, intensive care unit; CK-MB, creatine kinase-myocardial band; MI, myocardial infarction; STEMI, ST segment elevation myocardial infarction; NSTEMI, non-ST segment elevation myocardial infarction; IABP, intra-aortic balloon pump.

**Table 4:** In-hospital mortality according to medical treatment or IABP support.

	Medical (*n* = 105)	IABP < 2 h (*n* = 75)	IABP > 2 h (*n* = 12)	Medial vs. IABP < 2 h *p*-Value
In-hospital mortality	50 (47.6%)	24 (32.0%)	7 (58.3%)	0.036
ECMO implantation	24 (22.9%)	1 (1.3%)	3 (25.0%)	<0.001
In-hospital mortality	20 (83.3%)	1 (100.0%)	3 (100.0%)	1
Length of ICU stay	6.0 (2.0–14.0)	7.0 (4.0–12.0)	8.0 (5.0–12.5)	0.554
CRRT	27 (25.7%)	11 (14.7%)	3 (25.0%)	0.073
Stroke	1 (1.0%)	2 (2.7%)	0 (0.0%)	0.376
Vasoactive Inotropic Score	35.0 (16.5–90.0)	20.0 (8.4–60.6)	24.5 (20.0–45.7)	0.002

Values are *n* (%) or median (interquartile range). IABP, intra-aortic balloon pump; ECMO, extracorporeal membrane oxygenation; ICU, intensive care unit; CRRT, continuous renal replacement therapy.

## Data Availability

The data used during the current study is available from the corresponding author on reasonable request.

## Abbreviations

The following abbreviations are used in this manuscript:

AMI-CS	Cardiogenic shock due to acute myocardial infarction
CK-MB	creatine kinase-myocardial band
CRRT	Continuous renal replacement therapy
ECMO	Extracorporeal membrane oxygenation
IABP	Intra-aortic balloon pump
ICU	Intensive care unit
LM	Left main coronary artery
LVEF	Left ventricle ejection fraction
MCS	Mechanical circulatory support
MI	Myocardial infarction
STEMI	ST segment elevation myocardial infarction
LAD	Left anterior descending coronary artery
LV	Left ventricle
